# Deep Learning Image-Based Classification for Post-Earthquake Damage Level Prediction Using UAVs

**DOI:** 10.3390/s25175406

**Published:** 2025-09-02

**Authors:** Norah Alsaaran, Adel Soudani

**Affiliations:** Computer Science Department, College of Computer and Information Sciences, King Saud University, Riyadh 11543, Saudi Arabia

**Keywords:** deep learning, unmanned aerial vehicle (UAV), earthquake, MobileNet, convolutional neural network (CNN), real-time damage assessment, edge computing

## Abstract

Unmanned Aerial Vehicles (UAVs) integrated with lightweight deep learning models represent an effective solution for image-based rapid post-earthquake damage assessment. UAVs, equipped with cameras, capture high-resolution aerial imagery of disaster-stricken areas, providing essential data for evaluating structural damage. When paired with light eight Convolutional Neural Network (CNN) models, these UAVs can process the captured images onboard, enabling real-time, accurate damage level predictions that might with potential interest to orient efficiently the efforts of the Search and Rescue (SAR) teams. This study investigates the use of the MobileNetV3-Small lightweight CNN model for real-time post-earthquake damage level prediction using UAV-captured imagery. The model is trained to classify three levels of post-earthquake damage, ranging from no damage to severe damage. Experimental results show that the adapted MobileNetV3-Small model achieves the lowest FLOPs, with a significant reduction of 58.8% compared to the ShuffleNetv2 model. Fine-tuning the last five layers resulted in a slight increase of approximately 0.2% in FLOPs, but significantly improved accuracy and robustness, yielding a 4.5% performance boost over the baseline. The model achieved a weighted average F-score of 0.93 on a merged dataset composed of three post-earthquake damage level datasets. It was successfully deployed and tested on a Raspberry Pi 5, demonstrating its feasibility for edge-device applications. This deployment highlighted the model’s efficiency and real-time performance in resource-constrained environments.

## 1. Introduction

Earthquakes often lead to widespread structural damage, requiring immediate assessment for effective emergency response and recovery efforts. Among the various damages caused by earthquakes, building damage has the most significant impact on human life [1]. Traditional post-earthquake damage assessment methods involve manual inspection, which is time-consuming, resource-intensive, and hazardous in severely affected areas [2]. To overcome these challenges, the use of UAV technologies is being investigated by several research communities such as [3,4,5] for their potential applications in disaster area surveillance and victim localization. Modern UAVs offer significant advantages for these missions, as they are capable of transmitting high-resolution images, video, and other data in real-time from disaster zones. This data can be used to create detailed damage maps, thereby supporting decision-making for rescue teams and disaster management authorities. Additionally, they can be programmed for autonomous operation and are able to access locations that are challenging or hazardous for humans to reach [6].

In the context of post-earthquake building damage level prediction, deep learning models might play a key role in automating feature extraction and improving assessment accuracy. Factors such as type of the platforms and sensors, captured image quality, angles of the images, and types of the building damages, as noted in [7], directly affect how earthquake damage appears in images. Deep learning models, particularly CNNs, are well-suited to analyze and classify these variations in damage, even from complex and noisy data sources. CNN models can automatically detect patterns like cracks, displacements, and structural deformations, enabling more accurate and reliable assessments of damage levels. This ability to automatically extract and classify damage features ensures precise damage level assessments, which are critical for effective disaster response and resource allocation.

Autonomous UAVs rely heavily on onboard processing to perform tasks independently without transmitting data to a ground control station. However, this reliance on onboard processing introduces several challenges, including limited computational resources and limited embedded energy for system powering. Recent advancements in deep learning have led to the development of lightweight CNN models, which offer an efficient and effective approach to real-time processing on resource-constrained devices like UAVs. Models such as MobileNet [8] are designed to balance high performance with reduced computational complexity, making them potential candidates for onboard deployment in disaster level assessment. Additionally, deep learning approaches can surpass traditional machine learning methods, which rely on hand-crafted features, by utilizing transfer learning. In transfer learning, a pre-trained model is employed as a feature extractor, and additional layers are added on top to adapt the model for specific tasks. Moreover, fine-tuning is one of the most common techniques used in transfer learning to adapt a pre-trained model to a new task or dataset [9].

**Figure 1:**
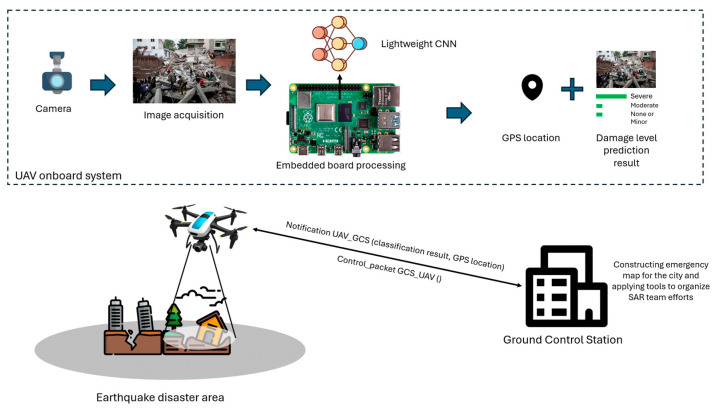
Application scenario for UAV-based real-time earthquake damage level classification using lightweight CNN models.

The specific use case addressed in this work involves a UAV (drone) equipped with a camera and an embedded system, deployed to assess earthquake damage along a predefined flight path. As the drone traverses the affected area, it captures images of buildings, infrastructure, and key locations. These images are processed in real time by a lightweight CNN model running on the UAV’s embedded system, which classifies the damage into three levels: none or minor, moderate, and severe. The classification is based on visual cues commonly used in post-earthquake damage assessment protocols. Specifically, none or minor damage includes superficial cracks, chipped plaster, or cosmetic wear that does not compromise structural integrity. Moderate damage involves more visible cracks in walls or columns, partial structural deformation, or localized failure of non-structural components. Severe damage refers to significant structural failures such as collapsed walls, exposed reinforcement, tilting, or total building collapse [10]. Each image is annotated with GPS coordinates, facilitating precise location tracking of the damaged areas. Upon completing the analysis, the results, along with the associated GPS data, are wirelessly transmitted to a Ground Control Station (GCS). This allows operators to monitor the situation in real time and receive alerts if severe damage is detected. A visual overview of this scenario is provided in Figure 1.

To achieve efficient real-time damage assessment, this work explores the use of pre-trained lightweight CNN models to predict post-earthquake damage levels, classifying the damage into three categories: none or minor, moderate, and severe. It focuses on the design of a lightweight deep learning model based on MobileNetV3-Small [11] intended for deployment on a UAV processing board. MobileNetV3-Small [11] is selected over other lightweight CNN architectures due to its superior trade-off between accuracy and computational efficiency on edge devices [12]. It incorporates architectural improvements like squeeze-and-excitation modules and hard-swish activation, which enhance feature extraction while maintaining a compact model size. Moreover, it offers a significantly lower number of Floating-Point Operations (FLOPs), which contributes to reduced computational cost and energy consumption, while still demonstrating promising performance in terms of accuracy compared to other lightweight CNN models such as EfficientNet-B0, ShuffleNet, and SqueezeNet [13,14].

To further enhance prediction accuracy, the adapted model utilizes transfer learning and fine-tuning. Transfer learning allows the model to leverage pre-trained networks, reducing the need for large labeled datasets and accelerating training. Fine-tuning adapts the model for the specific task of damage level classification, optimizing the final layers to improve performance on post-earthquake imagery. By combining MobileNetV3-Small with transfer learning and fine-tuning, the model achieves both accuracy and efficiency, making it well-suited for real-time damage assessment in resource-constrained environments. In order to validate the feasibility and performance of the adapted model, we perform an experiment on a Raspberry Pi 5. This allows for the evaluation of the model’s ability to run efficiently on a low-power, embedded system while maintaining a high level of accuracy in classifying damage levels from post-earthquake imagery. The experiment demonstrates the practical applicability of the model in real-world scenarios, confirming its potential for deployment on UAVs for rapid, on-site damage assessment in disaster-stricken areas.

The rest of this paper is organized as follows: Section 2 provides an overview of the underlying research field. Section 3 describes the MobileNetV3-Small-based architecture used for post-earthquake damage level prediction. Section 4 details the dataset, baseline models, experimental configuration, and evaluation criteria used to assess the performance of the adapted MobileNetV3-Small model. Section 5 presents and analyzes the evaluation results, while Section 6 discusses the deployment outcomes on Raspberry Pi 5. Finally, Section 7 summarizes the conclusions and future work.

## 2. Related Works

Real-time natural disaster classification is crucial for rapid emergency response, enabling timely and accurate decision-making in disaster-stricken areas. Kyrkou and Theocharides [13] addressed this challenge by introducing EmergencyNet, a highly efficient CNN model designed for emergency response applications. This model achieved near state-of-the-art accuracy (~95.7%) while offering a ~20× increase in computational speed, making it suitable for deployment on low-cost, low-power devices. They also presented the Aerial Image Database for Emergency Response (AIDER) dataset, aimed at aerial image classification in disaster scenarios. Their evaluation of various CNN architectures provides valuable insights into the trade-off between accuracy, inference speed, and memory efficiency aspects that directly inform the design choices in our lightweight model for UAV deployment. The AIDER dataset [13] has been used in various research studies for natural disaster classification, including the work by Lee et al. [15]. They proposed a computationally efficient model called Wider Attention EfficientNet (WATT-EffNet), which incorporated width-wise incremental feature modules and attention mechanisms. This focus on network width, rather than depth, is echoed in our model’s architectural strategy, which similarly leverages shallow but wide layers for maintaining performance under strict hardware constraints. The attention mechanisms further reduce computational costs by processing only key feature maps. Although this led to a slight increase in the number of training parameters, WATT-EffNet was shown to outperform established baselines in terms of F1 scores while using fewer FLOPs, demonstrating its computational efficiency. Yuan et al. [15] introduced EFFC-Net, which incorporates novel convolution blocks utilizing depthwise convolution [16] and group convolution [17], enhancing the network’s feature extraction capabilities while significantly reducing algorithm complexity. This design builds upon principles introduced in MobileNet, particularly the use of depthwise separable convolutions to achieve lightweight performance, making EFFC-Net similarly well-suited for deployment on resource-constrained devices. Shortcut connections were also used for feature fusion within the network. The performance of EFFC-Net was compared with advanced CNN and transformer models for disaster classification using the AIDER dataset [13], showing superior performance. Furthermore, Ijaz et al. [6] proposed a UAV-assisted edge computation framework that compresses CNN models for execution on an onboard embedded Graphics Processing Unit (GPU), enabling real-time classification of disaster scenarios. Their use of transfer learning to fine-tune pretrained models on small, domain-specific datasets inspired the training strategy adopted in our model, helping to improve generalization despite limited disaster-related data. They conducted an evaluation of various optimized TensorRT models on embedded GPUs, comparing latency, throughput, model size, and power consumption. TensorRT was applied with FP32 and FP16 precisions, with FP16 optimization demonstrating superior performance across these metrics. Among the models evaluated, MobileNet outperformed others in accuracy, loss, precision, number of parameters, and model size. This outcome reinforced our choice to adopt MobileNet as the backbone of our model, leveraging its efficient depthwise separable convolutions to maintain high accuracy while minimizing computational demands for UAV edge deployment. In terms of devices, the Jetson Xavier NX showed better performance in latency and throughput, while the Jetson Nano showed superior performance in power consumption. On the other hand, Shianios et al. [14] proposed a novel hybrid model, the Disaster Recognition Network (DiRecNetV2). The model combines the broad contextual strengths of Vision Transformer (ViT), which captures long-range dependencies within an image, with the local inductive biases of CNNs, which facilitate learning hierarchical features. Their model is specifically designed to meet the unique demands of UAV-based disaster management by integrating simple design choices such as depthwise convolution and a reduced number of heads and encoder blocks in the ViT. DiRecNetV2 was compared with lightweight CNN and ViT models using the AIDERSv2 dataset and a newly introduced multi-label dataset containing 300 images of overlapping disaster scenarios. The results demonstrated that the hybrid model, by combining CNNs and ViTs within a lightweight framework, offered promising solutions for deployment in resource-constrained environments, such as edge computing and mobile applications. The emphasis on lightweight design principles and efficient feature extraction in DiRecNetV2 aligns closely with the objectives of our model, which also targets deployment in resource-constrained disaster response environments.

Rapid and accurate assessment of building damage levels presents a significant challenge in post-disaster emergency response efforts. Zou et al. [18] proposed an enhanced YOLOv5s-Seg network for semantic segmentation in building damage classification from post-earthquake UAV imagery. The model improved the original YOLOv5s-Seg by incorporating the Mixed Local Channel Attention (MLCA) mechanism to reduce classification errors between adjacent building damage categories with similar textures. Additionally, they replaced the Neck part of YOLOv5s-Seg with the Neck from ASF-YOLO, a network designed for small target detection, which enhances the model’s ability to combine global and high-level semantic information from feature maps of different sizes. The results showed that the improved model outperformed existing methods, offering reduced model parameters, increased accuracy, and faster inference speeds, making it suitable for real-time rescue operations. This focus on reducing model parameters and boosting inference speed aligns with our objective in adopting MobileNet Small, which is also optimized for lightweight deployment and real-time performance on constrained devices.

Furthermore, Wang et al. [19] proposed a real-time method called DB-YOLOv5, which is based on YOLOv5 and designed for embedded systems to detect damaged building regions. They enhanced the YOLOv5 architecture in three ways: the network structure, feature fusion, and head of detection. The network structure of DB-YOLOv5 uses a residual dilated convolution (Res-DConv) model and removes the focus module of original YOLOv5, resulting in a lightweight network model suitable for deployment on embedded devices. The feature fusion combines spatial and channel attention mechanisms, effectively addressing the different scale problems of images during the UAV flight processing. Furthermore, the detection head utilized a fully connected head for classification and a convolutional head for bounding box regression, which improved both classification accuracy and localization precision. The results demonstrate satisfactory outcomes in the evaluation of UAV images used for building damage detection in the Wenchuan and Ludian earthquakes. Their approach reinforces the value of lightweight models for embedded systems—a direction also taken in our study by employing MobileNet Small to balance computational efficiency and detection performance.

Recently, Kizilay et al. [20] recently investigated the use of deep learning models for damage detection in drone imagery captured after the 2023 Hatay-Maraş earthquake. Their dataset addressed three tasks: a damage type dataset with a single class (collapsed), a damage level dataset with four classes (heavy, minor, moderate, and undamaged), and a wall damage dataset with two classes (vertical and horizontal). The authors explored the adaptation of models such as VGG16 for object detection via transfer learning, comparing their performance to established architectures like YOLOv8 and Detectron2. Their results highlighted YOLOv8’s superior performance across various evaluation metrics. Additionally, they applied OpenVINO [21] techniques, including quantization, to reduce model size and computational complexity. Yang et al. [22] proposed a real-time, anchor-free damage detection method called YOLOv6s-GRE. They implemented three key improvements: the introduction of the generalized feature pyramid network (GFPN) neck module, which enhanced damage detection accuracy; the design and integration of the Reparameterization Efficient Layer Aggregation Network (RepELAN) block, aimed at reducing both model size and computational complexity; and the development of an efficient detection head to accelerate detection speed while further decreasing model size and complexity. The YOLOv6s-GRE model was reconstructed using RepOptimizer (RepOpt) [23] to transform it into a quantization-friendly model, thereby addressing the quantization challenges associated with the reparameterization model. A partial quantization-aware training technique was employed achieving savings in memory and storage footprint while enhancing energy efficiency, making it suitable for deployment on UAVs.

Collectively, these studies highlight the value of attention mechanisms, lightweight convolutional designs, and model optimization strategies in developing efficient disaster assessment solutions. Building on these insights, our work adapts and fine-tunes MobileNetV3-Small for post-earthquake damage level prediction, with a focus on real-time performance, edge deployment, and classification accuracy within a UAV-based operational framework.

## 3. Design of the Classification Model

In this work, MobileNet [8] was chosen for the task of post-earthquake damage level prediction due to its efficiency and suitability for deployment in resource-constrained environments, such as embedded systems and mobile devices [24]. This section provides an overview of how MobileNet is applied to the prediction of building damage levels following an earthquake.

### Overview of MobileNet Architecture

MobileNet is a lightweight deep learning model developed by Google. It is designed to run efficiently on mobile and edge devices. This model is optimized for resource-constrained environments, striking a balance between high accuracy and low computational cost. The original architecture, MobileNetV1, was introduced in 2017. It uses depthwise separable convolutions to reduce computational complexity compared to standard convolutions, thereby achieving faster inference with fewer parameters while maintaining a high level of accuracy. Depthwise separable convolutions form the foundation of MobileNets. They split the standard convolution operations into two stages: Depthwise convolution, where filters are applied independently to each input channel, and Pointwise convolution, a 1 × 1 convolution that merges the outputs of the depthwise convolution. After the pointwise layer, batch normalization and ReLU6 are applied to each convolutional layer. ReLU6 [8] offers greater robustness compared to standard ReLU, preventing activations from becoming excessively large. MobileNetV2 [25] incorporates the use of inverted residuals and linear bottleneck layers, which enhance the model’s efficiency and lead to improved accuracy, particularly for tasks such as object detection and image classification.

MobileNetV3 [11] is optimized for deployment in both mobile and edge devices. It improves upon previous versions by leveraging enhancements in neural network design, such as Neural Architecture Search (NAS) [26], Squeeze-and-Excitation (SE) [27] modules, efficient convolution blocks, and Hard-Swish activation [11]. NAS is an automated technique for discovering the most effective neural network architecture for a specific task. In the case of MobileNetV3, NAS was employed to identify the optimal configuration of the network’s layers, filter sizes, and convolutional block structures. This process enabled the development of a highly efficient architecture that strikes a balance between accuracy and computational efficiency. As a result, MobileNetV3 is available in two primary variants: MobileNetV3-Large and MobileNetV3-Small, each tailored to different computational resources and performance requirements. MobileNetV3-Large is designed for higher accuracy and performance, suited for moderately powerful devices. MobileNetV3-Small is optimized for minimal computational resources and faster inference, ideal for low-power devices. The SE modules [27] contribute to improved model performance by adaptively recalibrating channel-wise feature responses. Specifically, they apply a lightweight attention mechanism that “squeezes” global spatial information into a channel descriptor and then “excites” each channel by weighting its importance, allowing the network to emphasize more informative features while suppressing less relevant ones. This improves feature representation and contributes to higher classification accuracy with minimal impact on model size and computational cost. Additionally, the use of Hard-Swish activation in MobileNetV3 offers the advantages of the Swish activation function, which provides improved performance over traditional ReLU, while being computationally more efficient and less resource-intensive.

**Figure 2:**
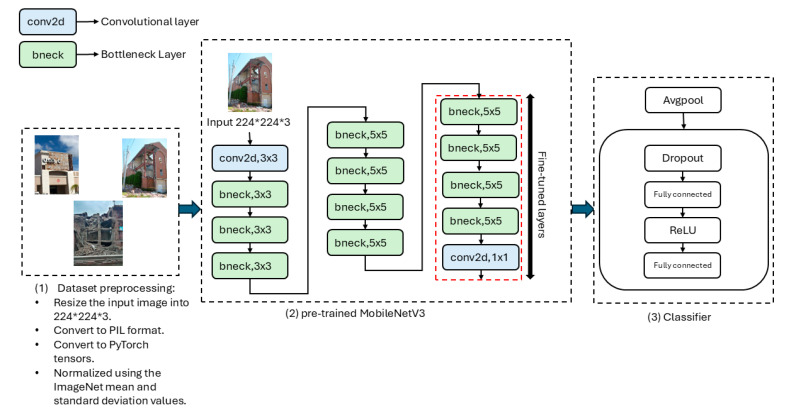
Architecture of the adapted MobileNetV3-Small model for post-earthquake damage level prediction.

In our approach, we used the pre-trained MobileNetV3-Small for the task of post-earthquake damage level prediction. The weights were initialized from training on the ImageNet dataset [28]. Initially, all parameters of the model were frozen, meaning that the pre-trained weights were not updated during the early stages of training. This strategy preserves the general feature extraction capabilities of the pre-trained model, while avoiding unnecessary computations in the initial phase. The final fully connected layers (classifier) of the MobileNetV3 model are then modified to adapt the network for the specific task of predicting damage levels in post-earthquake imagery. One widely used technique for model reuse is the fine-tuning approach, where a pre-trained model is further trained on a new, often smaller, dataset for a specific task. We have used this technique in order to leverage the knowledge the model has acquired from a large and diverse dataset, typically for a general task, and refine it for a specialized application. Furthermore, fine-tuning accelerates the learning process, reduces resource consumption, and enhances generalization to new tasks, making it a more practical solution than training models from scratch, especially when computational resources or training data are limited [9]. In the adapted MobileNetV3-Small model, fine-tuning was applied by unfreezing the last five layers of feature extractor, which are then updated during training to better learn domain-specific features. Additionally, the classifier part is also unfrozen, enabling it to be trained alongside the rest of the model. This approach allows the model to retain the general feature extraction capabilities of the pre-trained MobileNetV3 while adapting the final layers for the task at hand. By freezing most of the layers and only fine-tuning the last few, the model benefits from pre-learned feature representations, significantly reducing training time and computational costs. As shown in Figure 2, MobileNetV3-Small [11] architecture consists of 13 layers. The first layer is a convolutional layer (conv2d) that applies a lightweight depthwise separable convolution to the input image, extracting low-level features such as edges and textures. This layer is followed by 11 bottleneck layers (bneck) that use the inverted residual structure. They combine depthwise convolutions, pointwise convolutions, and linear bottleneck layers, enabling the model to efficiently capture hierarchical features while minimizing computational complexity. The Hard-Swish activation function is used in these layers to enhance non-linear representations, improving the model’s performance. After the 11 bottleneck layers, the architecture applies a final convolutional layer that performs a 1 × 1 convolution to aggregate the features learned by the previous layers. This layer combines information across the channels and refines the feature maps, acting as a compression step before the model transitions to the final phase. Following this, global average pooling (Avgpool) is applied to reduce the spatial dimensions of the feature maps, producing a compact representation of the learned features. Finally, a classifier layer is used to map the learned features to the output classes, predicting one of the three damage levels: none or minor damage, moderate damage, or severe damage.

## 4. Experimentation and Performance Evaluation of the Adapted Model

This section provides a detailed overview of the experimental setup used to evaluate the performance of the adapted model for post-earthquake damage level prediction. Section 4.1 presents an in-depth description of the dataset utilized in this study, including its characteristics, class distribution, and the preprocessing steps applied. Section 4.2 outlines the experimental configuration, detailing the hardware specifications, software frameworks, and relevant parameters employed during model training and testing. In Section 4.3, we describe the baseline models used for comparison with the adapted model. Finally, Section 4.4 discusses the evaluation metrics used to assess model performance, including accuracy, precision, recall, F1-score, and other relevant metrics, particularly suited for imbalanced datasets. Additionally, it discusses resource-related metrics that evaluate the efficiency of the model in utilizing computational resources.

### 4.1. Dataset

This work incorporates a merged dataset derived from three prominent sources: a Multi-Task Learning Dataset for Disaster Image Classification (MEDIC) [29], the Disaster Scenes Database [30], and Ünlü et al.’s [31] dataset. By combining these datasets, we aim to leverage their strengths to create a more diverse and comprehensive collection of images for the post-earthquake damage level prediction task.

MEDIC [29] is a large social media image classification dataset for humanitarian response, consisting of 71,198 images. It was used for four distinct tasks: The first task, disaster type classification, includes categories such as earthquake, fire, flood, hurricane, landslide, other disasters (e.g., plane or train crashes), and non-disaster images. The second task, informativeness classification, aims to filter out irrelevant images, with class labels comprising informative and non-informative. The third task, humanitarian classification, identifies images related to humanitarian efforts, with categories such as affected, injured, or deceased individuals; infrastructure and utility damage; rescue, volunteering, or donation efforts; and non-humanitarian images. Finally, the damage severity classification task categorizes images based on the severity of damage, with labels including severe damage, moderate damage, and minor or no damage. Another relevant dataset for disaster-related image analysis is the Disaster Scenes Database [30], which can be utilized for the task of post-earthquake damage level prediction. This dataset contains 1768 images and is designed to address two distinct tasks. The first task focuses on recognizing the contextual and causal properties embedded within the images, specifically identifying the hazard type. The second task involves estimating the damage level of an object, such as a building, within the image. The database includes three types of disaster scenes: tornado, tsunami, and earthquake. Additionally, the images are labeled according to the damage levels of built objects, which are categorized as no to minor damage, moderate damage, and severe damage. Moreover, Ünlü et al. [31] dataset consists of 900 images, with 300 images depicting normal buildings, 300 images depicting less damaged buildings, and 300 images showing damaged buildings. It is important to note that the dataset is specific to earthquake disasters and classifies images into three distinct categories: normal buildings, less damaged buildings, and severely damaged buildings.

The focus of this work is on predicting post-earthquake damage levels. Accordingly, several preprocessing steps are applied to both datasets to ensure that only images relevant to post-earthquake damage are included. In the MEDIC dataset [29], images labeled as ‘not disaster’, ‘fire’, ‘flood’, ‘hurricane’, ‘landslide’, or ‘other_disaster’ are removed. Additionally, images labeled with ‘affected_injured_or_dead_people’, ‘rescue_volunteering_or_donation_effort’, ‘not_informative’, or ‘not_humanitarian’ are excluded. Duplicate images were also removed from the dataset. For the Disaster Scenes Database [30], images representing other natural disasters such those labeled as ‘Tornado’ or ‘Tsunami’ were removed. The three datasets are combined and used to evaluate the adapted model, with the distribution of damage level classes across each dataset presented in Table 1.

In this study, we work with a dataset containing several imperfections that may affect the performance of the adapted model. These imperfections include mislabeling during data collection, which can lead to incorrect classifications, as well as images that are blurry, poorly lit, or captured from challenging angles, making it difficult for the model to extract relevant features. Additionally, the dataset contains outliers, such as images of irrelevant objects or scenes that do not accurately represent the intended class, introducing noise. To address these issues, approximately 30% of the dataset was manually cleaned according to the following criteria:

- Ambiguity threshold: Images were flagged as ambiguous if the visible damage could not be confidently categorized into one of the three levels (none or minor, moderate, severe) based on visible structural cues.
- Incorrect label correction: Labels were considered incorrect if the visible damage level contradicted the definition criteria. For example, an image labeled as “severe” but showing only superficial wall cracks was relabeled as “minor.”
- Outlier removal: Images were removed if they showed no relevant buildings or earthquake-related structures (e.g., people, or vehicles) or were taken from extreme angles that obscured damage assessment.

Examples of cleaned data include replacing a “moderate” label with “severe” for an image showing large structural cracks and exposed reinforcement and discarding images showing interior scenes unrelated to structural damage, such as furniture or debris piles without structural context.

After this initial manual cleaning, the model was incrementally trained on the refined subset. It was then used to assist in classifying the remaining 70% of images. The newly classified images were reviewed, incorporated into the dataset, and went through further manual checks to ensure accuracy. The details of these updates, such as the number of images that were added, moved, or removed, are summarized in Table 2.

**Figure 3:**
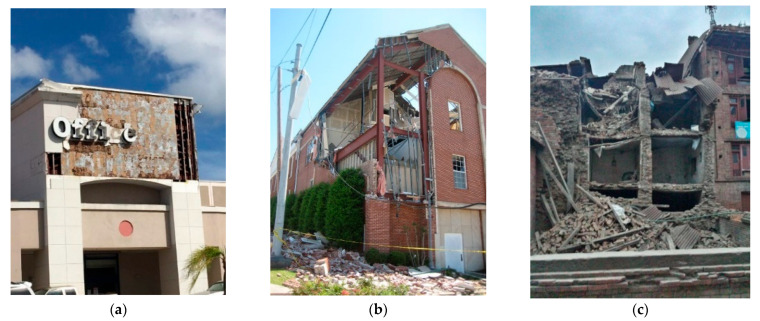
Sample of images in the dataset, each representing a different damage level: (**a**) none or minor damage, (**b**) moderate damage, and (**c**) severe damage.

As shown in Table 2, the dataset used in this study is imbalanced, with the majority class (severe damage) significantly outnumbering the other classes. To mitigate this issue, StratifiedShuffleSplit [32] was used to ensure that the class distribution in the training, validation, and test sets reflects the original distribution of the dataset. This technique helps maintain a proportional representation of both the majority and minority classes, thereby providing more reliable performance metrics and ensuring that the model is evaluated effectively across all classes. The distribution of damage levels across the training, testing, and validation datasets is illustrated in Table 3. Figure 3 shows a sample of images in the dataset, each representing a different damage level. All images have a horizontal resolution of 96 dpi, a vertical resolution of 96 dpi, and each pixel is represented by 24 bits.

### 4.2. Experimentation Setup

The experimentation of the adapted model and its evaluation were conducted using an NVIDIA GeForce RTX 4070 Laptop GPU and an Intel^®^ Core™ i9-185H 2.50 GHz processor, running on the Windows 11 operating system. The deep learning framework used was PyTorch 2.5.1, with CUDA 11.8 for GPU acceleration. The training dataset was employed for model training, while the validation dataset was used to select the optimal hyperparameters. The specific hyperparameter values used for the models are presented in Table 4.

### 4.3. Baseline Models

Five baseline models are used to assess the performance of the adapted model: MobileNetV2 [25], MobileNetV3-Small [11], SqueezeNet [33], ShuffleNetV2 [34], and EfficientNet-B0 [35]. These models were selected due to their lightweight architectures and proven effectiveness in classification tasks, particularly in resource-constrained environments such as mobile and embedded devices. SqueezeNet [33] is a lightweight CNN designed to achieve competitive performance with a significantly smaller model size. Its innovation lies in the use of fire modules, consisting of a squeeze layer (1 × 1 convolutions) followed by an expand layer (1 × 1 and 3 × 3 convolutions), which reduces the number of parameters without compromising accuracy. This makes SqueezeNet well-suited for applications with strict memory and computational resource limitations. On the other hand, ShuffleNetV2 [34] improves upon the original ShuffleNet design by incorporating optimizations such as channel shuffling and group convolutions. These techniques reduce computational complexity, improve processing speeds, and minimize memory usage while maintaining high accuracy. ShuffleNetV2 is particularly effective for large-scale image classification tasks on mobile and embedded devices where efficiency and speed are crucial. Moreover, EfficientNet-B0 [35], the smallest model in the EfficientNet family, uses a compound scaling method to balance depth, width, and resolution, optimizing both accuracy and efficiency. Its efficient design makes it suitable for high-accuracy tasks with limited computational resources. For all base line models, the pre-trained versions were utilized. The final fully connected layer, or classifier, was set to output the required number of classes for post-earthquake damage level prediction. All layers, except the classifier, were frozen to prevent their weight from being updated during training. Only the classifier layer was fine-tuned on the new dataset, which is efficient as the pre-trained layers have already learned useful features.

### 4.4. Evaluation Measures

In the case of imbalanced datasets, relying on accuracy may not provide a comprehensive assessment of the model’s performance. To offer a more detailed evaluation, several accuracy-related metrics were used. These include accuracy, precision, recall, F1-score, confusion matrix, and Receiver Operating Characteristic (ROC) curve. Equation (1) defines accuracy as the ratio of correctly predicted instances to the total number of instances. Equation (2) defines precision as the ratio of correctly predicted positive observations to the total predicted positives. Equation (3) defines recall as the ratio of correctly predicted positive observations to all actual positive instances. Equation (4) defines the F1-score as the harmonic mean of precision and recall, offering a single metric that balances both measures, which is particularly useful in scenarios with imbalanced class distributions.

(1) $$Accuracy=\frac{TP+TN}{TP+TN+FP+FN}$$

(2) $$Precision=\frac{TP}{TP+FP}$$

(3) $$Recall=\frac{TP}{TP+FN}$$

(4) $$F1-Score=2*\frac{Precision*Recall}{Precision+Recall}$$

where TP, FP, and FN refer to true positives, false positives, and false negatives, respectively. Moreover, the confusion matrix [36] is used to evaluate the model’s performance. It allows for a comprehensive assessment of the classification performance by showing how well the model’s predictions align with the true class labels. It is particularly useful in classification tasks, especially for imbalanced datasets, as it goes beyond simple accuracy metrics and helps identify specific areas where the model may be making errors. In multi-class classification, it is often useful to calculate a weighted average of the F1-scores across all classes, especially when the classes are imbalanced. Equation (5) defines the weighted average F1-score. N is the number of classes, ${F1-Score}_{i}$ is the F1-score for class i, and ${support}_{i}$ is the number of true samples of class i in the dataset.

(5) $$Weighted\;Average\;F1-Score=\frac{\sum_{i=1}^{N}\left({support}_{i}*{F1-Score}_{i}\right)}{\sum_{i=1}^{N}{support}_{i}}$$

ROC curves graphically represent the performance of a binary classification model by plotting the True Positive Rate (TPR) against the False Positive Rate (FPR) at various threshold values. The Area Under the Curve (AUC) measures the model’s ability to distinguish between positive and negative classes, with values ranging from 0 to 1, where higher values indicate better performance. For multiclass classification, this work employed the One-vs-Rest strategy, treating each class as the positive class and the others as negative, resulting in a ROC curve and AUC score for each class.

The loss function used in this work is Cross-Entropy loss function [37]. For a batch of N samples, the total loss is computed as the average loss across all samples as shown in Equation (6) where $y_{ni}$ is the true label (one-hot encoded) for sample n and class i, $p_{ni}$ is the predicted probability for sample n and class i, and C is the total number of classes. To address class imbalance, Compute_class_weight [38] is used in conjunction with Cross-Entropy loss to assign higher penalties for misclassifying the minority class. This increases the loss for errors in predicting the minority class, encouraging the model to focus more on correctly classifying these instances. Consequently, the model is less biased toward the majority class and better able to accurately identify the minority class.

(6) $$loss=-\frac{1}{N}\;\sum_{n=1}^{N}\sum_{i=1}^{C}y_{ni}log(p_{ni})$$

To evaluate a post-earthquake damage level prediction model, particularly in resource-constrained environments such as mobile devices or embedded systems, it is crucial to consider resource-related metrics that assess how efficiently the model utilizes computational resources during both training and inference. Key metrics include FLOPs, which represent the number of floating-point operations required during inference and indicate the computational complexity of the model [39]; Model Size, referring to the number of learnable parameters; and Memory Usage, which quantifies the amount of memory consumed during training and inference. Moreover, Frames Per Second (FPS) is a metric that quantifies the number of individual frames (images) a model can process per second. It is commonly used to assess the performance of models, particularly in tasks such as image classification [13]. FPS is influenced by several factors, including batch size, hardware, and model efficiency. By evaluating these resource-related metrics, it is possible to ensure that the model is not only performing well in terms of accuracy-related metrics but also optimized for deployment in environments with limited resources, balancing efficiency and effectiveness.

## 5. Results and Discussion

To obtain a reliable evaluation of the adapted model, it was trained five times, and the average accuracy, precision, recall, and F1-score were calculated across these runs. This approach mitigates the impact of random weight initialization, which is inherent in neural networks and can introduce variability in performance.

The evaluation results for the three damage levels identified by the adapted model are presented in Table 5. As shown, the model demonstrates varying performance across the three damage levels. For the ‘None or Minor’ class, the model achieves a precision of 0.84 and a recall of 0.86. In contrast, the ‘Moderate’ class shows lower precision and recall values of 0.60 and 0.64, respectively. Although the recall for the ‘Moderate’ class is slightly higher than its precision, both metrics remain relatively low compared to the ‘None or Minor’ class. This indicates the model struggles to accurately distinguish between these two categories, with somewhat greater difficulty in correctly classifying moderate damage. One likely factor contributing to this underperformance is the limited number of samples in the ‘Moderate’ class (953 images), which restricts the model’s ability to learn representative features of moderate damage. This data imbalance may cause the model to confuse moderate damage with either minor or severe classes, reducing both precision and recall. For ‘Severe’, the model performed the best, achieving high precision (0.97) and recall (0.96), suggesting it is quite effective at identifying severe damage instances. The overall weighted F1-score of 0.93 reflects the model’s good overall performance, balancing its ability to classify severe damage with some challenges in the minority classes. The accuracy of 0.93 indicates that the model correctly classifies 93% of all instances, but the discrepancies in class-specific performance highlight the need for improvement in handling the minority classes, particularly the ‘None or Minor’ and ‘Moderate’ categories.

**Figure 4:**
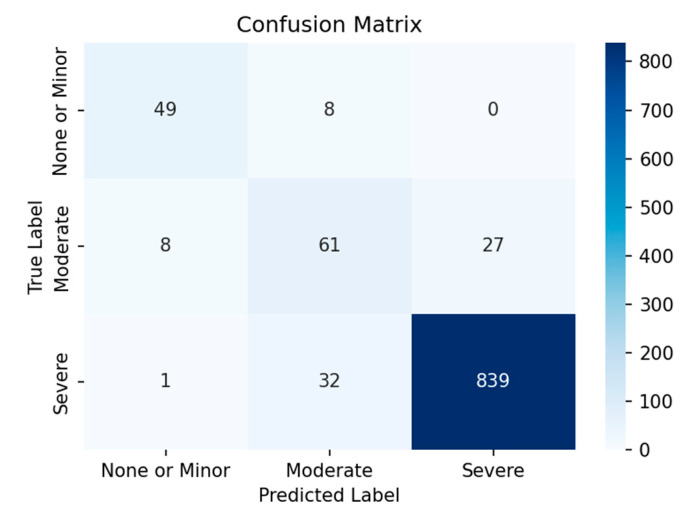
Confusion matrix of the adapted model.

Figure 4 presents the confusion matrix obtained by MobileNetV3 with fine-tuning the last 5 layers. As illustrated, the model demonstrated difficulty in accurately classifying samples from the ‘none or Minor’ and ‘moderate’ classes, while performing significantly better on the ‘Severe’ class. This is due to the presence of a class imbalance, where the ‘severe’ class is overrepresented in the dataset. As a result, the model has likely become biased towards the ‘severe’ class, leading to better performance on it while struggling with the other classes. This issue persists despite the implementation of techniques designed to address class imbalance.

**Figure 5:**
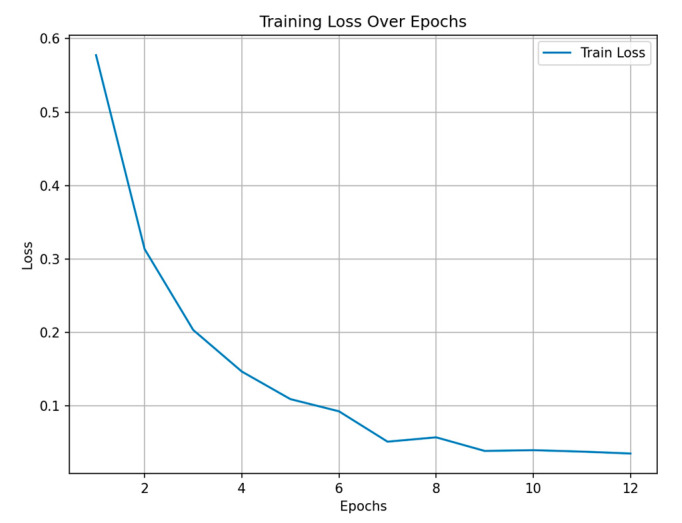
Training loss over epochs of the adapted model.

Figure 5 illustrates the training loss over epochs. As shown, the training loss decreased substantially during the initial stages of training, particularly between epochs 1 and 8, indicating effective learning and model optimization. However, after epoch 8, the loss curve began to plateau, with no significant reduction in loss observed in the subsequent epochs. This trend suggests that the model had reached a point of convergence and that further training was unlikely to yield meaningful performance improvements. This early convergence can be attributed, in part, to the use of a pre-trained model, which provided a well-initialized set of parameters, enabling the network to adapt more rapidly to the target task. As a result, the model required fewer epochs to reach optimal performance compared to training from scratch. Consequently, an early stopping mechanism was employed, and training was terminated at epoch 12. The application of early stopping not only prevented potential overfitting but also improved training efficiency by halting the process once diminishing returns were evident.

**Figure 6:**
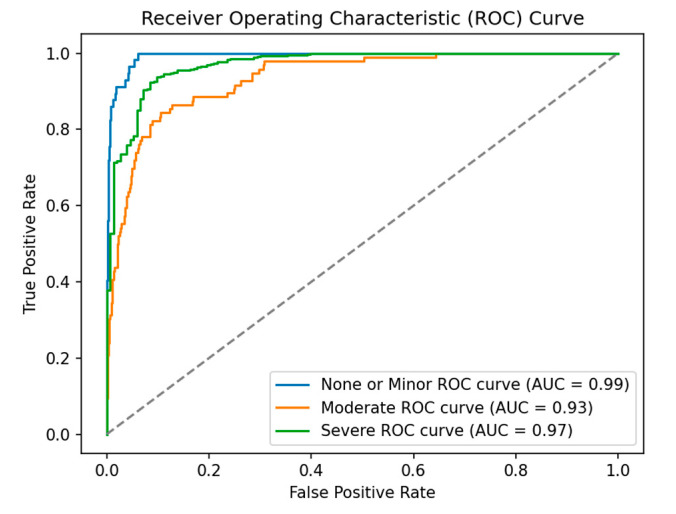
ROC curves and AUCs for MobileNetV3 finetuned model.

Figure 6 presents the ROC curve and AUC for each class. As shown, the ‘None or Minor’ class achieves an AUC of 0.99, indicating that the model is effective in distinguishing instances of this class from others. However, given that the ‘None or Minor’ class is underrepresented relative to the other classes in the dataset, the model is able to learn the general distinctions between classes well, resulting in a high AUC. Despite this, the model may still face challenges in making accurate individual predictions for this class due to the class imbalance. This difficulty in correctly identifying instances of the underrepresented class can lead to poor precision and recall, which consequently lowers the F1-score. The ‘Moderate’ class achieves an AUC of 0.91, suggesting that the model encounters a moderate number of false positives and false negatives within this category. As a result, the model’s ability to effectively distinguish instances of the ‘Moderate’ class is less pronounced compared to the ‘None or Minor’ and ‘Severe’ classes. For the ‘Severe’ class, the model achieves an AUC of 0.97, which indicates that the model is effective at correctly identifying severe damage cases.

Overall, the model shows strong overall performance, especially for the ‘None or Minor’ and ‘Severe’ classes, where the AUC scores are both high (0.99 and 0.97, respectively). The AUC for ‘Moderate’ (0.93) is lower, suggesting that the model may face more difficulty in distinguishing between moderate damage and the other categories. This is likely due to the nature of the ‘Moderate’ class images, which may have features that overlap with both the ‘None or Minor’ and ‘Severe’ classes. For example, moderate damage image might include partial structural issues, which could appear similar to the intact or minimally damaged features of a ‘None or Minor’ class image. At the same time, these images may also share some similarities with severely damaged buildings, particularly in areas where the damage is more pronounced but not complete. In contrast, images of non-damaged buildings (‘None or Minor’) and severely damaged buildings have more distinct visual features, such as intact structures or highly deteriorated ones, making them easier to differentiate. This overlap in features for the ‘Moderate’ class leads to challenges for the model, resulting in a relatively lower AUC score and highlighting the difficulty in accurately classifying this category.

**Figure 7:**
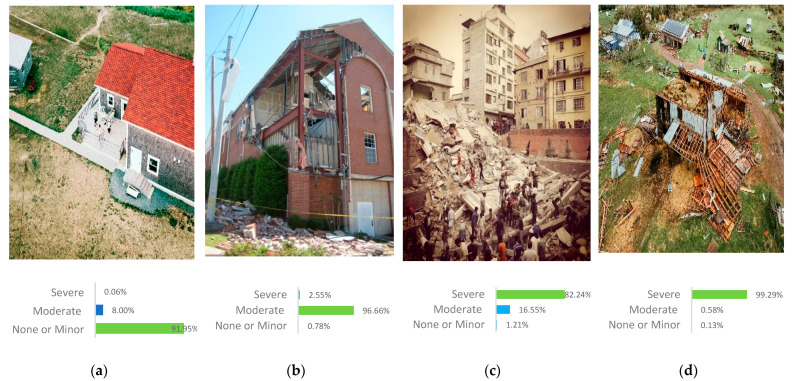
The examples demonstrate the adapted model proficiency in identifying post-earthquake damage level. (**a**) None or Minor damage example, (**b**) Moderate damage example, (**c**,**d**) Severe damage examples.

Figure 7 presents the predictions obtained by the adapted model for identifying post-earthquake damage, using a subset of four images from the test set. These images demonstrate the model’s strong classification performance across three categories: None or Minor damage, Moderate damage, and Severe damage. Figure 7c illustrates an intriguing performance of the model in classifying the damage level of the building. The model classifies the image as having severe damage with a high probability of 82.24%. This classification is consistent with the image’s context, where the building appears predominantly affected by significant damage. While moderate damage is also evident in some parts of the building (16.55%), the model identifies severe damage as the most prominent feature.

To evaluate the model’s performance, the adapted MobileNetV3+ fine-tuning model was compared against several baseline models. As shown in Table 6, MobileNetV3+ finetuning achieves superior results in terms of the overall weighted average F1-score. However, all models encountered challenges in accurately classifying the ‘None or Minor’ class, with F1-scores ranging from 0.67 to 0.82. Among the models, MobileNetV3+ fine-tuning demonstrated the best performance for this class, achieving an F1-score of 0.82. For the ‘Moderate’ class, all models exhibited comparable F1-scores, ranging from 0.42 to 0.63, which is notably lower than the performance observed for the ‘Severe’ class. The most significant difference is observed in the ‘Severe’ class, where the MobileNetV3+ fine-tuning model achieved a high F1-score of 0.97, outperforming the other models, which achieved scores ranging from 0.91 to 0.94. Additionally, when comparing the overall F1-scores of MobileNetV3 with fine-tuning of the last five layers (denoted as MobileNetV3+ fine-tuning) to MobileNetV3 with only the classifier finetuned, it is evident that finetuning the last five layers results in a 0.04-point improvement in overall F1-scores. This suggests that fine-tuning the last layers of the model contributes to enhancing its overall performance.

To evaluate the efficiency of the model in computational resource requirements, the results of resource-related metrics are presented in Table 7. In particular, the number of FLOPs is an important metric, as models with higher FLOPs tend to exhibit slower inference times, which can become a bottleneck, particularly in real-time applications. As indicated in the table, MobileNetV3 achieved the lowest number of FLOPs, demonstrating superior efficiency in terms of computational resources. MobileNetV3+ fine-tuning, however, comes close, with a slight increase of approximately 0.1 in the FLOPs. Despite this marginal increase in computational complexity, MobileNetV3+ fine-tuning outperformed MobileNetV3 by 0.04 points in weighted average F1-score, indicating a trade-off between computational cost and model performance. In terms of the number of parameters, the SqueezeNet model has the fewest trainable parameters and the smallest model size.

To the best of our knowledge, this is the first contribution that uses the MEDIC dataset [29], the Disaster Scenes Database [30], and Ünlü et al.’s [31] dataset for training a post-earthquake damage level prediction model. To evaluate the performance of the adapted model, a comparison is made with the model proposed by Tang et al. [40]. In their work, the authors proposed three models: Faster R-CNN with a ZF feature extractor, Faster R-CNN with ResNet5, and the Single Shot Multibox Detector (SSD). Among these, Faster R-CNN with ResNet5 achieved the best performance. To compare the adapted post-earthquake damage level prediction model with the Faster R-CNN with ResNet5 model from [40], the experiment is reconducted using the Disaster Scenes Database [30] (discussed in Section 4.1). Their model was trained using all three types of natural disasters, as well as all three levels of damage severity. As a result, their model is not specific to earthquake disasters. Tang et al. in [40] used approximately 85% of the dataset for training and 15% for testing. The experiment was conducted with the same dataset distribution to ensure consistency. Moreover, the test dataset used in [40] consisted of 61 examples of the None or Minor class, 113 examples of the moderate class, and 80 examples of the severe class. The test dataset is constructed to mirror the class distribution of the test dataset used in [40], ensuring an equal representation of each damage level. This approach preserves balance and enables a fair comparison across the different damage categories. Table 8 presents a comparison between the adapted model and the Faster R-CNN with ResNet-50 model [40]. As shown, the adapted model outperforms the Faster R-CNN with ResNet-50 model by 0.02 in F1-score. The Faster R-CNN with ResNet-50 model requires 240,000 epochs for training, whereas the adapted model achieves comparable performance after only 28 epochs. This highlights the advantage of using a pre-trained model, such as MobileNetV3, over training a model from scratch.

Furthermore, a comparison is made with the model proposed by Ünlü et al. [31], which was based on three pre-trained CNNs: VGG16, VGG19, and NASNet. This study addressed two post-earthquake image classification tasks: classifying images into two categories (damaged buildings and normal) and classifying images into three categories (damaged buildings, less damaged buildings, and normal). In contrast, our study focuses on the classification of images into three levels of damage. To ensure a fair comparison, we retrain the adapted model following the same dataset distribution used by Ünlü et al. [31], which allocates 70%, 15%, and 15% of the dataset for training, validation, and testing, respectively. Also, ensuring that the test set contains 44 samples from the damaged category, 45 from the less damaged category, and 47 from the normal category. Additionally, the VGG19 model is re-implemented as described in [31] to compute the model’s FLOPs, number of parameters, and overall model size. As shown in Table 9, the adapted model outperforms the results achieved by the model presented in [31] in terms of accuracy, FLOPs, number of parameters, and model size. This indicates that the adapted model not only achieves higher performance but also utilizes computational resources more efficiently. As such, it offers a more scalable solution for post-earthquake damage assessment tasks, making it better suited for deployment in onboard systems where resource constraints are a critical consideration.

## 6. Deploying the Model on Raspberry Pi

**Figure 8:**
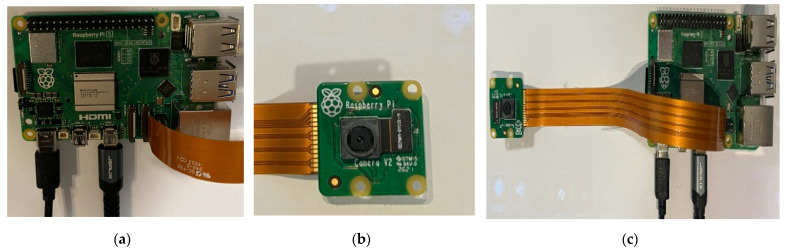
Experimental setup: (**a**) Raspberry Pi 5 Module, (**b**) Pi camera module, and (**c**) shows the connection of Pi camera on the Raspberry Pi Module.

This section evaluates the deployment of the adapted model on a Raspberry Pi 5 for the classification of images into three levels of damage. The Raspberry Pi 5, equipped with 8 GB of RAM and a 64-bit quad-core Cortex-A76 CPU, provides a balance of computational power and energy efficiency, while maintaining a compact form factor that is ideal for use in UAVs. The device was configured with the Raspbian OS and integrated with essential libraries, such as PyTorch for model inference, OpenCV for image preprocessing, and other necessary Python 3.12.3 libraries. Additionally, a camera module was connected to the Raspberry Pi, enabling the capture of real-time images, which were subsequently processed by the model for classification. Figure 8 illustrates the experimental setup employed in this study.

**Figure 9:**
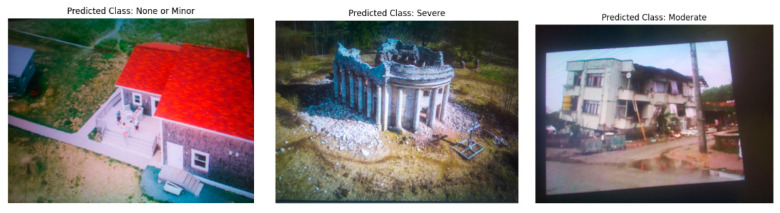
Some comparison examples of prediction results generated by the adapted model after deploying on Raspberry Pi5.

To evaluate the performance of the adapted model on the Raspberry Pi 5, key metrics such as FPS and average inference time were measured. Table 10 presents a comparison of these metrics across different models. Notably, SqueezeNet achieves the highest FPS value, though the performance of the other models remains comparable. While SqueezeNet outperforms MobileNetV3+ fine-tuning by 1.9 FPS, MobileNetV3+ fine-tuning demonstrates a slight advantage in terms of weighted average F-score, outperforming SqueezeNet by 0.06 points. This analysis provides valuable insights into the trade-offs between computational speed and classification accuracy, particularly in embedded systems for real-time UAV applications. Figure 9 shows the predictions generated by the adapted model when deployed on the Raspberry Pi 5 for post-earthquake damage identification, using randomly selected images from the test set.

Moreover, to assess the performance of the adapted model after its deployment on the Raspberry Pi 5, we reimplement one of the lightweight models discussed in related research, namely EmergencyNet by Kyrkou et al. [13]. This model is employed for the task of predicting damage levels. As shown in Table 11, our model demonstrates superior performance compared to EmergencyNet [13] in terms of the weighted average F-score, achieving a value of 0.93. This indicates a better balance between accuracy, precision, and recall. However, our model exhibits lower processing speed, with a FPS rate of 3.2, in contrast to EmergencyNet’s FPS of 6.11. Additionally, our model has a longer average inference time per image (0.3117 s) compared to EmergencyNet [13], which is 0.1638 s. These results suggest that, while our model achieves higher accuracy, it is less efficient in terms of processing speed.

## 7. Conclusions

This paper investigates the use of lightweight CNNs for real-time post-earthquake damage level prediction using UAV-captured imagery. Providing real-time information on the extent of the damage is crucial for emergency response teams, as it allows them to quickly assess the affected areas and prioritize their efforts. This timely data enables faster decision-making, allowing SAR teams to reach the region more effectively and potentially save many lives by delivering aid and resources to the most impacted zones. In this study, MobileNetV3-Small, a model optimized for resource-constrained environments, is used to efficiently assess earthquake damage. The adapted model classifies the damage into three distinct levels: none or minor, moderate, and severe damage. The model was trained and evaluated on a merged dataset of three dedicated earthquake datasets, which exhibits a highly imbalanced distribution of damage level classes. To address this imbalance, various techniques such as class weighting were applied during the training process. Several baseline models were evaluated to benchmark the performance of the adapted model, including MobileNetV2, SqueezeNet, ShuffleNetV2, and EfficientNet-B0.

Experimental results demonstrate that MobileNetV3-Small achieves the lowest number of FLOPs and ranks second in terms of the fewest parameters and smallest model size among the baseline models. It achieves a 58.8% reduction in FLOPs compared to the baseline model with the lowest FLOPs. Fine-tuning the last five layers of MobileNetV3-Small resulted in a slight increase in resource-related metrics but led to notable improvements in accuracy-related metrics. The overall accuracy gains across different severity levels ranged from 3.20% to 18.90%, indicating a modest to moderate improvement in performance compared to the baseline MobileNetV3 model. Although the FLOPs saw a slight increase (of 0.2%), this change is negligible. To further assess the model’s overall performance, it was retrained and evaluated on a natural disaster classification task, yielding promising results. The adapted model offers a scalable solution for post-earthquake damage assessment, capable of operating on low-power embedded platforms like Raspberry Pi and NVIDIA Jetson, making it ideal for deployment in disaster zones. Future work can explore further optimizations to improve model robustness in varying environmental conditions and enhance the overall efficiency of the UAV-based assessment system.

## Figures and Tables

**Table 1:** Damage level class distribution in MEDIC [31] dataset, Disaster Scenes Database [32], and Ünlü et al. [33] dataset.

Dataset	None or Minor	Moderate	Severe	Total
MEDIC [29]	333	1200	8124	9657
Disaster Scenes Database [30]	127	389	242	758
Ünlü et al. [31] dataset	300	300	300	900
Total	760	1889	8666	11,315

**Table 2:** Dataset Update Overview—The number of newly added images, reassigned images, and removed images are detailed.

Dataset	None or Minor	Moderate	Severe	Total
Original dataset	760	1890	8676	11,315
Removed images	153	258	937	1348
Moved images	Images moved to moderate: 70 Images moved to severe: 216 Total moved image: 286	Images moved to none or minor: 14 Images moved to severe: 883 Total moved image: 897	Images moved to none or minor: 8 Images moved to moderate: 105 Total moved image: 113	1296
New images	240	52	152	444
New dataset	583	962	8877	10,424

**Table 3:** Train, validation, and test dataset statistics for each damage level class.

Damage Level	Train	Validation	Test	Total
None or Minor	458	57	57	572
Moderate	763	95	95	953
Severe	6974	872	872	8718
Total	8195	1024	1024	10,243

**Table 4:** Experimental environment.

Parameter	Value
Learning rate	1 × 10^−4^
Input size	224 × 224
Batch size	32
Weight decay	1 × 10^−5^
Dropout	0.2
Optimizer	Adam

**Table 5:** Accuracy-related metrics of post-earthquake damage level prediction MobileNetV3+ finetuning model.

	None or Minor	Moderate	Severe	Overall
TP	49	61	839	949
TN	959	889	126	1974
FP	9	40	27	76
FN	8	35	33	76
Precision	0.84	0.60	0.97	0.93
Recall	0.86	0.64	0.96	0.93
F1-score	0.85	0.62	0.97	Weighted Average F1-Score: 0.93
Accuracy	-	-	-	0.93

**Table 6:** Accuracy-related metrics of post-earthquake damage level prediction model and baseline model.

Model	None or Minor F1-Score	Moderate F1-Score	Severe F1-Score	Weighted Average F1-Score
MobileNetV2	0.80	0.55	0.94	0.90
MobileNetV3	0.76	0.53	0.94	0.89
SqueezeNet	0.80	0.52	0.93	0.87
ShuffleNetv2	0.69	0.42	0.91	0.85
EfficientNet-B0	0.67	0.43	0.91	0.85
MobileNetV3+ finetuning (**our model**)	**0.82**	**0.63**	**0.97**	**0.93**

**Bold** indicates the best results.

**Table 7:** Resource-related metrics of post-earthquake damage level prediction model and baseline model.

Model	FLOPs × 10^6^	Number of Parameters (M)	Memory Usage (MB)
MobileNetV2	326.2	2.20	8.50
MobileNetV3	**61.5**	1.50	5.80
SqueezeNet	262.9	**0.70**	**2.76**
ShuffleNetv2	151.7	1.30	4.79
EfficientNet-B0	413.9	4.00	15.30
MobileNetV3+ finetuning (**our model**)	61.6	1.65	6.29

**Bold** indicates the best results.

**Table 8:** Comparison between the adapted model and the Faster R-CNN with ResNet5 model by Tang et al. [40].

Model	Weighted Average F1-Score	None or Minor F1-Score	Moderate F1-Score	Severe F1-Score	Epoch
Faster R-CNN with ResNet5 model by Tang and Chen [40]	0.63	**0.71**	0.57	0.62	240,000
Our	**0.65**	0.64	**0.63**	**0.67**	**28**

**Bold** indicates the best results.

**Table 9:** Comparison between the adapted model and Ünlü et al.’s [31] VGG19 model.

Model	Accuracy	FLOPs	Parameters	Model Size (MB)
VGG19 by Ünlü and Kiriş [31]	0.66	6,489,657,344	139,582,531	532.47
Our	**0.73**	**61,585,400**	**1,649,443**	**6.29**

**Bold** indicates the best results.

**Table 10:** Comparison of FPS and Average inference time per image between the adapted model and other lightweight models.

Model	MobileNetV3+ Finetuning	MobileNetV2	MobileNetV3	SqueezeNet	ShuffleNetv2	EfficientNet-B0
FPS	3.21	2.63	3.31	5.11	3.86	2.51
Average inference time per image (seconds)	0.3117	0.3805	0.3021	0.1955	0.2594	0.3982

**Table 11:** Performance comparison of EmergencyNet by Kyrkou [13] and our adapted model.

Model	Weighted Average F-Score	FPS	Average Inference Time per Image (Seconds)
EmergencyNet by Kyrkou et al. [13]	0.87	**6.11**	**0.1638**
Ours	**0.93**	3.21	0.3117

**Bold** indicates the best results.

## Data Availability

The original data presented in the study are openly available in [29,30,31].
